# Remarks on the Application of Poultices to Inflamed Eyes

**Published:** 1847-02

**Authors:** 


*Remarks on the application of Poultices to Inflamed Eyes.* By C. T. Quintard, M. D., *Assistant Physician to Bellevue Hospital.*

In the second nnmber of the Annalist, I notice some excellent remarks by Dr. Dubois, on the impropriety of applying poultices to the inflamed eye. After stating the opinions of a number of distinguished surgeons on the subject, the Doctor states, that his experience perfectly agrees with that of the gentlemem whose opinions he quotes. The following cases, which has come under my notice, convince me of the excellence of the Doctor's remarks.

P. McC., of good constitution—and whose general health had always been of the best—entered the Hospital on the 12th of August last, his right eye presenting the following appearances: Lids highly inflamed and swollen, between which the cornea, completely disorganized, was bulging, and exhibited signs of sloughing. At the inner canthus there was a not very copious pnrulent discharge. He informed me that about a week previous, he had a "bad feeling" in the eye, accompanied with frequent shooting pain through the orbit. That by the advice of a "Dutch doctor," he applied a warm hop fomentation over the eye, and followed it with a warm bread and milk poultice; and although this to a certain degree assuaged the pain, the lids, nevertheless, continued swelling, and on the second or third day after the application of the poultice, his sight was entirely lost. As nothing could be done on his admission to the Hospital to restore either the beauty or the usefulness of the organ, I directed my remedies to the reduction of the inflammation. If the fatal result of this case was not the effect of the application of the warm poultices, we presume they, in some measure, contributed to it; and although they may relieve pain, we feel confident they generally are prejudicial, and never so happy in their effects as other and more active remedies. From what have come under our notice, we presume this mode of treating ophthalmic inflammations, is more common than surgeons in private practice generally suppose.

Passing for an "old woman's remedy," which is thought to do no harm, even if it does no good, the poultice is applied, and while the pain is diminished the eye is destroyed. No practitioner, but the most thoughtless or ignorant, would rashly meddle with so exquisitely delicate an organ as the eye, or recommend any lotion, unguent or fomentation, without inquiring minutely into the difficulty, and having some data upon which to found his reasoning and practice.

It is admitted by the most eminent ophthalmic surgeons, that poultices are in very few cases beneficial, and that the good effects to be derived from them, may be attained by other means. We conclude, therefore, that it is safer at all times to try other remedies, before we subject a patient to treatment of such doubtful propriety.

J. W. entered the eye wards, with the lids of the right eye very much swollen, and the cornea nearly of the same aspect as in the former case. Warm poultices had been applied, followed by the same results as we have described above. He stated that, three days after applying poultices to the right eye, the left became affected, but by the use of cups, blisters, and a lotion prescribed by a physician, the inflammation ceased. These are two of four cases which I have witnessed, while I have been an assistant physician at Bellevue Hospital, in which the destruction of the eye has resulted from a practice we must consider empirical.—*N. Y. Annalist.*

